# Spinal Orthosis in Adolescent Idiopathic Scoliosis: An Overview of the Braces Provided by the National Health Service in Italy

**DOI:** 10.3390/medicina60010003

**Published:** 2023-12-19

**Authors:** Cristina Maria Del Prete, Domiziano Tarantino, Mattia Giuseppe Viva, Massimiliano Murgia, Daniele Vergati, Giovanni Barassi, Eleonora Sparvieri, Eugenio Di Stanislao, David Perpetuini, Emanuele Francesco Russo, Serena Filoni, Raffaello Pellegrino

**Affiliations:** 1 Department of Physical Medicine and Rehabilitation, ASL LE, 73100 Lecce, Italy; cridelprete@tin.it; 2 Department of Public Health, University of Naples Federico II, 80131 Naples, Italy; domiziano22@gmail.com; 3 Department of Anatomical and Histological Sciences, Legal Medicine and Orthopedics, Sapienza University of Rome, 00183 Rome, Italy; mattiagiuseppe.viva@uniroma1.it (M.G.V.); massimiliano.murgia@uniroma1.it (M.M.); 4 Orthopedic Workshops Orthogea, 72017 Ostuni, Italy; medicalvergati@libero.it; 5 Center for Physiotherapy, Rehabilitation and Re-Education-CeFiRR-Gemelli Molise, 86100 Campobasso, Italy; giovanni.barassi@unicatt.it; 6 Department of Internal Medicine, ASL Teramo, 64100 Teramo, Italy; 7 Orthopedic Workshops I.T.O.P., 00036 Palestrina, Italy; distanislao.e@itop.it; 8 Department of Engineering and Geology, University “G. d’Annunzio” of Chieti-Pescara, 65127 Pescara, Italy; david.perpetuini@unich.it; 9 Padre Pio Foundation and Rehabilitation Centers, 71013 San Giovanni Rotondo, Italy; emanuele.fr88@gmail.com; 10 I.R.R.C.S. Casa Sollievo della Sofferenza, 71013 San Giovanni Rotondo, Italy; serena.diba@gmail.com; 11 Department of Scientific Research, Campus Ludes, Off-Campus Semmelweis University, 6912 Lugano–Pazzallo, Switzerland; raffaello.pellegrino@uniludes.ch; 12 Santa Chiara Institute, 73100 Lecce, Italy

**Keywords:** adolescent idiopathic scoliosis, conservative treatment, bracing treatment, braces

## Abstract

Adolescent idiopathic scoliosis (AIS) is a lateral, rotated curvature of the spine. It is a 3-dimensional deformity that arises in otherwise healthy children at or around puberty. AIS is the most common form of scoliosis in the pediatric population. The etiology is multifactorial, including genetic and environmental factors. The incidence is roughly equal between males and females, while there is a higher risk of progression in females. Guidelines for AIS treatment identify three levels of treatment: observation, physiotherapy scoliosis-specific exercises, and braces. In this paper, we carried out a review of the scientific literature about the indication and success rates of the braces provided for free by the National Health Service in Italy (SSN). Despite a general consensus on the efficacy of rigid bracing treatment and its use in AIS, an important heterogeneity about the treatment is present in the scientific literature, demonstrating a high degree of variability. The overall success rate of the braces provided by the SSN is high, suggesting an important therapeutic role in the treatment of AIS. Robust guidelines are needed to ensure uniform and effective treatments.

## 1. Introduction

Adolescent idiopathic scoliosis (AIS) is the most common form of all scoliosis in the pediatric population (80%) [1,2] and consists of a lateral, rotated curvature of the spine. It is a 3-dimensional deformity that affects healthy children around puberty. Ponseti et al. distinguished four major types of scoliosis: thoracic, lumbar, thoraco-lumbar, and S-shaped [3].

The etiology is multifactorial, including genetic and environmental factors [4]. According to different studies, the prevalence of AIS ranges from 0.47% to 5.2% in the general population [5,6]. At least 10% of those with AIS will require treatment, and 0.1–0.3% will require surgical correction [5,7]. The progression of AIS is much more frequent in females, especially in severe scoliosis [4]. 

The diagnosis of AIS is defined when other causes of scoliosis, such as neuromuscular diseases, syndromes, and malformations of the spine, have been excluded [1,8,9]. Adam’s Forward Bending Test, the gold standard clinical test for scoliosis screening (sensitivity of 84.37% and specificity of 93.44%) [10], allows us to differentiate scoliosis into two categories: I) dysmorphism postural disfunction (Adam’s Forward Bending Test positive), which involves an element of permanent rotation within the spine and ribcage in addition to the lateral curvature; and II) paramorphism (Adam’s Forward Bending Test negative), in which postural or other asymmetries mimic scoliosis and its symptoms [11]. 

The natural history of scoliosis was described by Weinsten et al. [12,13], and to quantify the risk of progression, several methods have been developed. Of these, the most used is the one proposed by Lonstein and Carlson, which includes the Cobb angle, Risser sign, and chronological age in the evaluation [14]. The Cobb angle is one of the most important parameters when assessing curve severity and is measured on a posteroanterior X-ray spine film. A curve with a Cobb’s angle less than 20 degrees is classified as mild. A moderate curve is between 25 degrees and 40 degrees. A severe curve is more than 40 degrees [15,16]. The instrumental diagnosis of AIS based on a frontal curve of the spine is greater than 10° Cobb, as measured on a postero-anterior (P-A) spine radiograph (X-ray) [17]. 

The six-point Risser system is used to classify the stage of bone maturation since it was noticed how the progression of scoliosis occurs, especially during the phase of greater growth, and then the curve stabilizes at the end of growth. Risser stated that the attachment of the iliac apophysis could be used as a marker of the completion of vertebral growth, so its classification is based on the amount of excursion of the iliac apophysis [17]. 

A conservative rehabilitative approach is first-line treatment for AIS. Several therapeutic exercises have been studied in the literature, such as aerobic and resistance training [18], Schroth exercises [3,19], and different scoliosis schools’ approaches to physiotherapy scoliosis-specific exercises (PSSE) [20,21,22]. The goals are either morphological (stop or even reduce the curve progression at puberty and improve aesthetics) or functional (prevent or treat respiratory dysfunction and prevent or treat spinal pain syndromes) [23]. 

The most recent guidelines of the International Scientific Society on Scoliosis Orthopedic and Rehabilitation Treatment (SOSORT, 2016) [24] and the recent review by Alison et al. (2021) [1] have rigorously indicated the operative modalities of the rehabilitation treatment of patients with AIS; furthermore, they have highlighted the important role of bracing in the rehabilitation process, considered perceptual and not just as a mechanical value. The SOSORT guidelines, published in 2018, marked an important point in the evidence on conservative treatment for idiopathic scoliosis due to the presence of emerging high-quality evidence, allowing the formulation of some grade-I recommendations about the effectiveness of PSEE and bracing [24]. Some findings were outlined by the guidelines of the Scoliosis Research Society (SRS) for AIS treatment, identifying three levels of treatment: observation, PSSE, and braces [25]. Halsey et al. in 2021 assessed the percentage of concordance with the SOSORT Guidelines of AIS specialists, finding that the large majority (99% of the sample) use braces in the treatment of AIS, and almost 90% of them refer to the SRS criteria in bracing prescription [26]. A brace is a mechanical system that effectively counteracts a mechanical problem. The mechanism of action is based on the application of external forces; the purpose is to redistribute mechanical stress to promote vertebral remodeling, reduce scoliosis, and aim to restore axiality. Bracing a scoliotic curve unloads the growth plates on the concave side of the vertebral bodies near the curve’s apex. Growth stimulation, leading to structural remodeling of the vertebral bodies on the curve’s concave side, may explain the improvement or lack of curve progression reported with successful brace management of AIS [27,28]. The response of the scoliosis curve to the action of a brace and the reactivity of the viscoelastic structures allows the remodeling of the curve itself, and the effectiveness of this process is inversely proportional to the skeletal age of the patient. 

The goal of brace treatment is to prevent further progression of the AIS and to prevent the need for surgery [29]. Early diagnosis and early bracing could decrease the risk of spine surgery [24]. Unfortunately, most AIS patients present too late for effective management with braces [1,24]. Brace treatment should be reserved for curves above 20 ± 5° Cobb and residual growth periods. Curves below 15 ± 5° Cobb should not be placed in orthotic treatment. Part-time bracing or night-time bracing has been described by some physicians and is widely advocated in some institutions. There is a lack of long-term follow-up results to prove the effectiveness of this type of treatment in AIS, and all series on effective orthotic treatment are with full-time wear [30,31].

In 2013, a multicenter randomized clinical trial (RCT) on patients with suggestions for bracing considering age, skeletal immaturity, and degree of scoliosis was performed by Weinstein et al. [13]. The analysis included patients randomly assigned into two groups: a treatment group, which underwent a bracing treatment (for at least 18 h/day), and an observational control group. The bracing treatment did not improve the outcome of the progression of curves greater than 50 degrees, while it was effective in the progression of curves of lesser degrees. In addition, there was a significant association between hours of brace wearing and treatment success. Therefore, the authors concluded that bracing prevents the need for surgery [13]. Although there is consensus on the effectiveness of bracing treatment, a high degree of variability in the clinical approach and prescription is present. These include, for example, the prescribed hours, the frequency of follow-up and X-ray examinations, the use of the brace at night, and the times of suspension and weaning. Since a variation in these parameters could make the difference between a therapeutic success and a therapeutic failure, SOSORT and the SRS agreed that there is a need for standardization of research methods to ensure more effective evidence.

However, it is worth highlighting that the effectiveness of braces for scoliosis treatment is multifaceted. In fact, while braces are generally recommended for adolescents with idiopathic scoliosis to halt curvature progression [32], their effectiveness is limited in certain cases. For instance, in children with scoliosis complicated by spinal cord injury, bracing may not prevent surgery or may delay the need for surgical correction as the curve size increases [33]. Additionally, a randomized controlled trial found no significant difference in preventing curve progression for moderate scoliosis between bracing and scoliosis-specific exercises [34]. Furthermore, the success of stabilizing or improving scoliosis through bracing is modest, often requiring virtually continuous brace wear and resulting in low compliance [35]. Moreover, the type of brace used can impact its effectiveness, with hard braces being considered more effective than soft braces for scoliosis treatment [36]. In this perspective, Niu et al. developed a robotic brace that is able not only to apply active corrective forces to the spine but also to support rehabilitation exercises, indicating the potential for robotic technology to assist in the conservative management of spinal deformities [37]. Notably, while the direct application of robotic assistive training [38,39] (for scoliosis treatment) is not extensively documented in the literature, the broader evidence on the use of robotic technology in musculoskeletal rehabilitation and orthopedic interventions suggests its potential utility in the management of scoliosis, as corroborated by the effectiveness of the robotic brace developed by Niu et al. [37].

However, for adolescents with severe idiopathic scoliosis, alternative treatments such as vertebral body tethering or surgical intervention may be necessary if the curves continue to progress despite bracing [40]. It is important to note that physical therapy plays a key role in preventing scoliosis curve progression and improving outcomes and quality of life through manual therapy, bracing, core stabilization, and strengthening [41]. With the aim of strengthening back muscles, transcutaneous electrical nerve stimulation (TENS) has been explored as a potential treatment for scoliosis. While some studies have dismissed electrical stimulation as a viable treatment for scoliosis [42], others have suggested its potential to induce muscle contraction and improve back asymmetry in subjects with scoliosis [21]. Additionally, it has been proposed that TENS can excite muscle spindles or Golgi tendon organs to induce proprioceptive illusions, similar to the approach of mechanical vibration [43]. Furthermore, TENS has been widely used to induce muscle contraction in both clinical neurorehabilitation and laboratory settings [44]. In the context of scoliosis, lateral electrical surface stimulation (LESS) has been found to be effective in improving trunk balance in children with severe cerebral palsy and scoliosis [45]. Importantly, TENS can also be delivered through wearable devices, such as suits equipped with electrodes able to administer customized electrical stimulation [46]. The effectiveness of these suits to improve trunk control was demonstrated in children affected by cerebral palsy [47].

This paper aims to provide an overview of the scientific literature regarding the commonly used braces that are included in the Essential Levels of Care (LEA) of the Italian National Health Service (SSN) and provided to citizens free of charge in terms of indications and success rate [48].

## 2. Materials and Methods

All the procedures related to this overview were organized and reported after performing a search in the main scientific electronic databases (PubMed, Scopus, and Web of Science) to identify the available scientific articles about the braces included in the LEA of the SSN, with no restriction of time. Only articles in English were reviewed.

The search was performed by three independent authors (CM.DP, D.T, and R.P.) at their own institutions, and two independent reviewers (D.T. and R.P.) extracted and evaluated the data from the selected articles. For the purposes of our review, the following keywords were used alone or in combination: adolescent idiopathic scoliosis, scoliosis, conservative treatment, bracing. 

Only articles regarding the braces included in the LEA of the SSN were selected, regardless of the type of article. The information about the braces provided by the SSN was directly extracted from the institution’s website [48].

To facilitate the understanding of the results, the braces were reported as single sub-sections with specific information about each one. Given the nature of our work, we neither performed a systematic search nor reported the results in a systematic review fashion.

## 3. Results

### 3.1. Standard Rigid Braces

**Figure 1:**
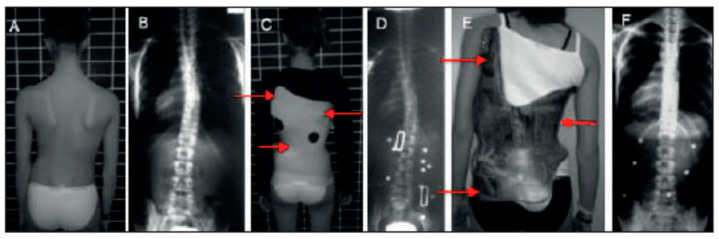
Three-point pressure brace principle. (**A**) clinical assessment of the scoliosis; (**B**) radiographic evaluation; (**C**) brace derotation point of action; (**D**) radiological assessment of the brace; (**E**) definitive brace; (**F**) radiological assessment during the follow-up.

The principle that determines the mechanism of action of the main spinal orthoses is that of three-point pressure (Figure 1), which aims at the correction of deformities with traction, lateral deflection, and derotation. If made correctly, a brace can drive vertebral growth and stop the evolution of scoliosis. There is a wide variety of braces to choose from; the choice depends on the location and rigidity of the curves, the age, and specific patient preferences [30].

### 3.2. Milwaukee Brace

**Figure 2:**
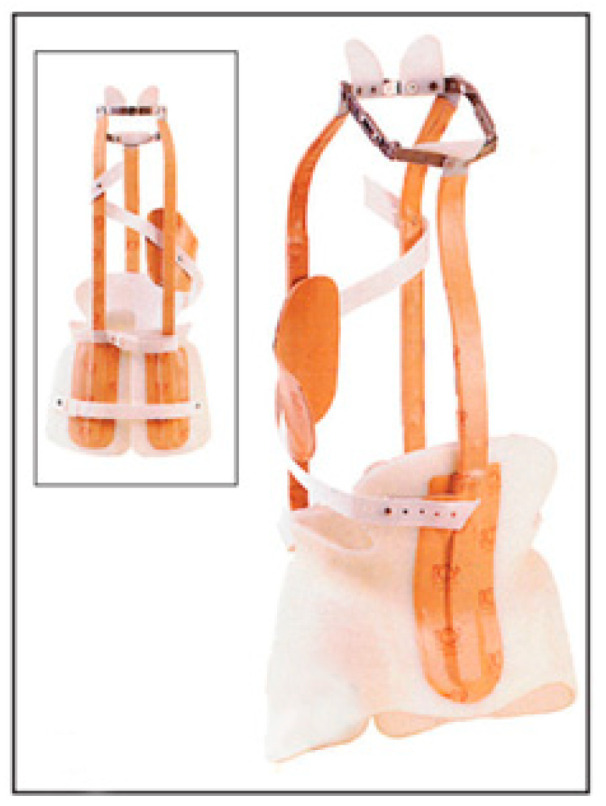
Milwaukee brace.

The Milwaukee brace (Figure 2) was developed in 1954 for the conservative treatment of idiopathic scoliosis; it is contraindicated for mature individuals. It consists of a neck ring and a pelvic mold that fix and extend the spine, inducing constant self-elongation. An axillary sling and thoracic and lumbar pads apply transverse corrective forces to the curves, resulting in a consequent correction of the deformity [49].

The corrective mechanism is based on the elongation of the trunk and the opening of the convexities of the curves through the thrusts located on the ribs afferent to the apex of the curves to carry out a derotation of the vertebral body through the ribs. In this type of brace, the three-point system has a less important role in the final outcome, as the primary mechanism of action is to stimulate the muscles of the trunk, allowing a redirection of spinal growth [50].

The indications for the Milwaukee brace treatment include the following [51]:

1. Mild and moderate curves, up to 40–45 degrees, during the skeletal growth period;
2. In cases of severe curves, it is more effective than other braces in upper-thoracic and cervical curves, and it is the only brace recommended for proximal curves up to D5;
3. Patients require less chest restriction and more ventilation and comfort.

The use of the Milwaukee brace for curves between 20° and 29° with a Risser sign between 0 and 1 showed less progression (28%) than untreated curves of similar magnitude. When using the Milwaukee brace for curves of similar magnitude but with a Risser sign of 2 or more, the progression rate is 10% less than for untreated curves. Similarly, curves between 30° and 39° with a Risser sign between 0 and 1 progressed 14% less using the Milwaukee brace than untreated curves of similar magnitude, and treated curves of similar magnitude, but a Risser sign of 2 or more progressed 21% less than untreated curves [14,52]. The rate of success of this kind of brace ranges from 43% to 53.33% [53,54].

Among the side effects of this brace, the most relevant are the psychological impact due to its neck ring [55] and the induction of a flat back. Today, in clinical practice, it has apparently been replaced by TLSOs (thoraco-lumbo-sacral orthoses), such as the Boston brace and the Cheneau brace. Studies comparing the Milwuakee and the Boston braces showed that the Boston brace was more successful than the Milwaukee brace irrespective of initial curve magnitude and skeletal maturity, especially for lumbar and thoracic scoliosis [55,56]. The Cheneau brace was shown to provide better spinal decompensation as well when compared to the Milwaukee brace [57].

### 3.3. Boston Brace

**Figure 3:**
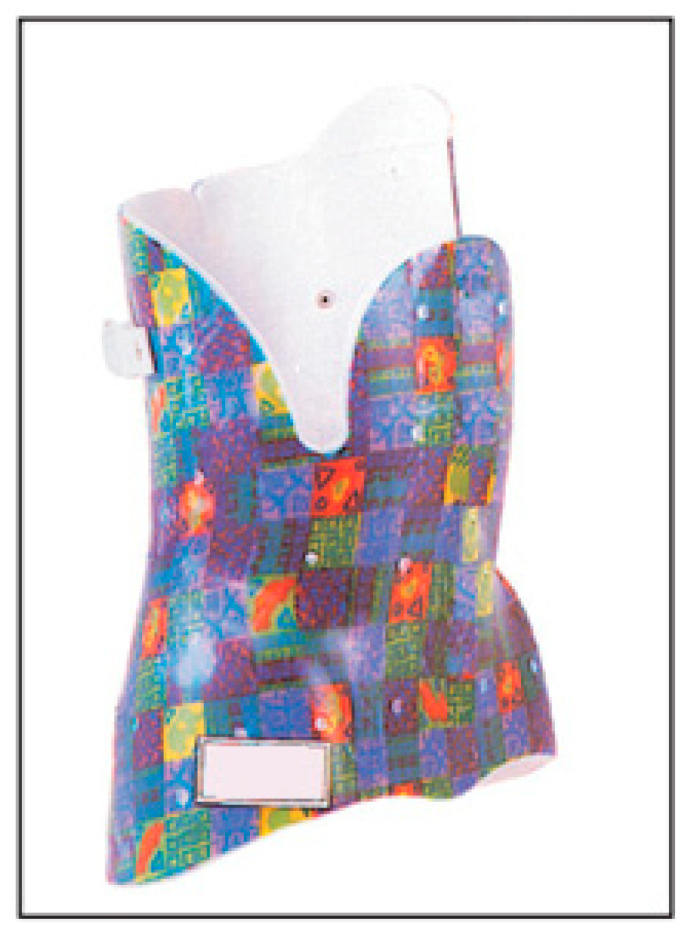
Boston brace.

The Boston brace (Figure 3) was designed by Hall and Miler in 1972 [58], and its efficiency was first reported by Watts et al. in 1977 [59]. It is a prefabricated polypropylene brace, open on the back, subsequently personalized for the patient. The correction of the curve on the coronal plane and the vertebral rotation take place through pads added to complete the brace that push the spine, giving a rotational force to the ribs and increasing the upright position of the spine [60,61]. The biomechanical action produced by the Boston brace allows, through the project performed on radiographic examination, the placement of thrusts capable of derotating the vertebral bodies. The main goal is to correct the lateral deviation and rotation of the vertebral bodies, bringing the trunk back into trim on the sacrum.

The TLSO Boston brace is indicated as follows:

4. Lumbar and thoracolumbar or thoracic (below T8) curves between 20 and 50 Cobb degrees. In 2013, a RCT by Weinstein et al. [13] found that pediatric patients with idiopathic scoliosis who wore Boston braces had a 72% success rate of achieving a Cobb angle of <50 degrees, against the 48% in the observation (control) group.
5. Similar curves greater than 50 Cobb degrees exist in patients who cannot undergo surgery for clinical reasons.

The aim of the Boston brace is a correction of at least 50%, so the permanent correction two years after brace discontinuance can be 15% in relation to the initial angle [30]. Using the Boston brace, 49% of the curves remain unchanged, 39% of the scoliosis has a permanent correction of 5° to 15°, 4% are stabilized with a correction of at least 15°, 4% lose between 5° and 15°, and 3% progress more than 15° [30]. A study by Emans et al. described that 11% of patients treated by Boston had a surgical indication during the period of bracing [58].

### 3.4. Cheneau Brace

**Figure 4:**
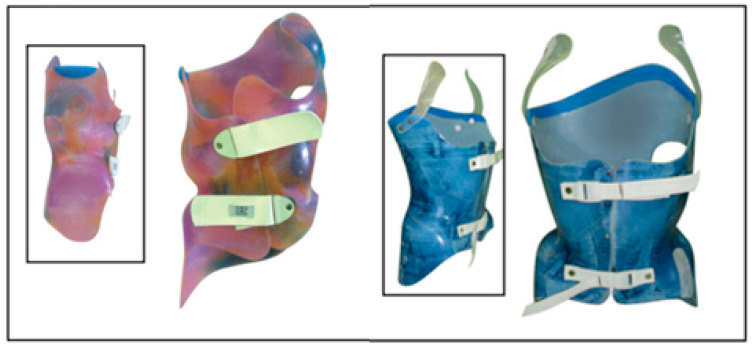
Cheneau brace.

Jacques Chêneau designed the original Chêneau brace in 1979 [30]. The brace (Figure 4) is fabricated in polypropylene and has an anterior opening with Velcro. It is a lightweight and well-accepted brace that corrects in the three planes of space with good effectiveness on rotation. The Cheneau brace is asymmetrical, used for patients of all degrees of severity and maturity, and often worn for 20 to 23 h/day [30]. The brace principally aims to allow lateral and longitudinal rotation and movement [62]. The aim of the Chêneau brace is to obtain a three-dimensional correction of the scoliotic curve. The detorsion on the sagittal plane would also lead to normalization on the coronal and transverse planes and a consequent elongation of the spine without the application of distractive forces. According to Cheneau, the deformation of the scoliotic body consists of the following: 1. convexities and concavities; 2. sagittal configuration deformity; 3. rotation of the pelvis and shoulders; and 4. lateral displacement. The corset acts through a three-dimensional, three-point system, allowing it to reduce humps, mobilize the flat parts, and leave respiratory movements free. It provides for a triple action: active, passive, and dynamic. It corrects the deformity without favoring the flat back that is commonly associated with scoliosis. The biomechanical principle is to apply thrusts on the convex side of the curves and on all the humps detectable on the trunk, both front and rear, and develop large expansion chambers where there are depressions and on the concave side of the curves.

The indication for the Cheneau brace is progressive AIS with an apex lower than T5 and a Cobb angle between 25° and 45° [63]. 

One study showed that the Cobb angle was reduced by an average of 16.4° [64], and two studies reported a success rate (defined as no more than 5 degrees of curve progression) of 76% and 81% using, respectively, a Rigo Cheneau brace and a modified Cheneau (called “Cheneau-P”) [65,66], while another study reported a success rate (defined as preventing curve progression beyond 50 degrees) of 81% [67].

Some studies demonstrate how Cheneau brace correction could not only influence the progression of the scoliotic curve, but also its natural history [68,69,70], even if there is no consensus on its efficacy. A retrospective study comparing the Cheneau to the Boston brace outlined that the Cheneau led to a lower mean and percent major curve progression than the Boston brace [71]. The use of different criteria for outcome assessment could at least partially explain the lack of agreement in the literature. Most commonly, failure is defined by the need for surgery or by a certain amount of radiographic curve progression during treatment or follow-up, while standardized criteria are needed [72].

### 3.5. Lyon Brace

**Figure 5:**
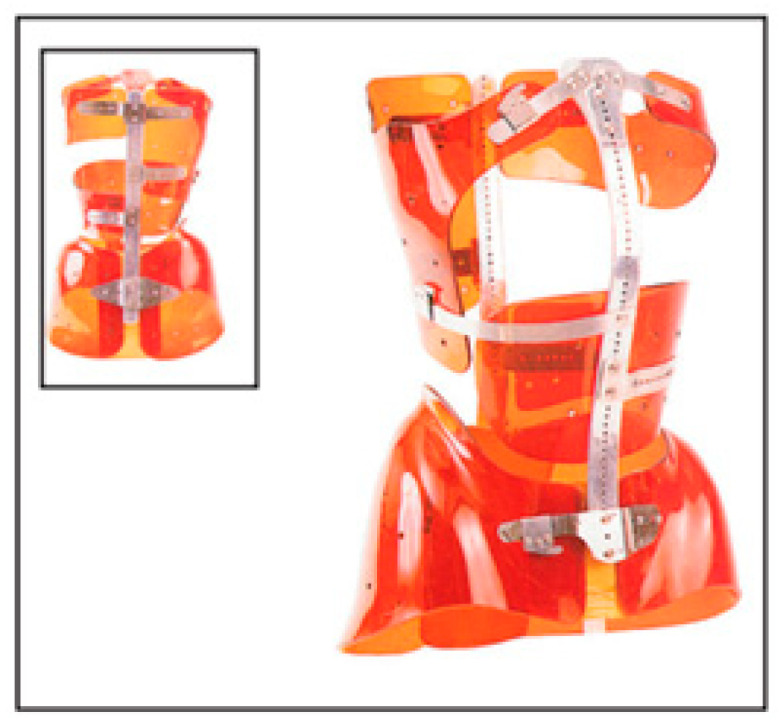
Lyon brace.

The Lyon brace was invented by Stagnara et al. in 1947 and is also known as the Stagnara brace. This brace system is the first to have prospective studies and consistent documented efficacy [30]. The Lyon is a rigid brace (Figure 5) used to treat lumbar or low thoracolumbar scoliotic curves between 30 and 50 Cobb degrees. Can also be treated with Lyon brace curves greater than 50 Cobb degrees that cannot be operated on for medical reasons or skeletal immaturity. It is also prescribed after surgical treatment for 3 to 6 months. The principles of Lyon brace are based on the three-point pressure theory, with an application of a force on the two neutral vertebras and a counterforce at the apex of the curve. Derotational forces are also applied on the spine, from the posterior convexity to the anterior concavity [73]. The overall efficacy of the Lyon brace is 95%. However, it drops to 87% for thoracic curves and to 80% in patients with Risser sign 0 [30].

### 3.6. Very Rigid Braces

**Figure 6:**
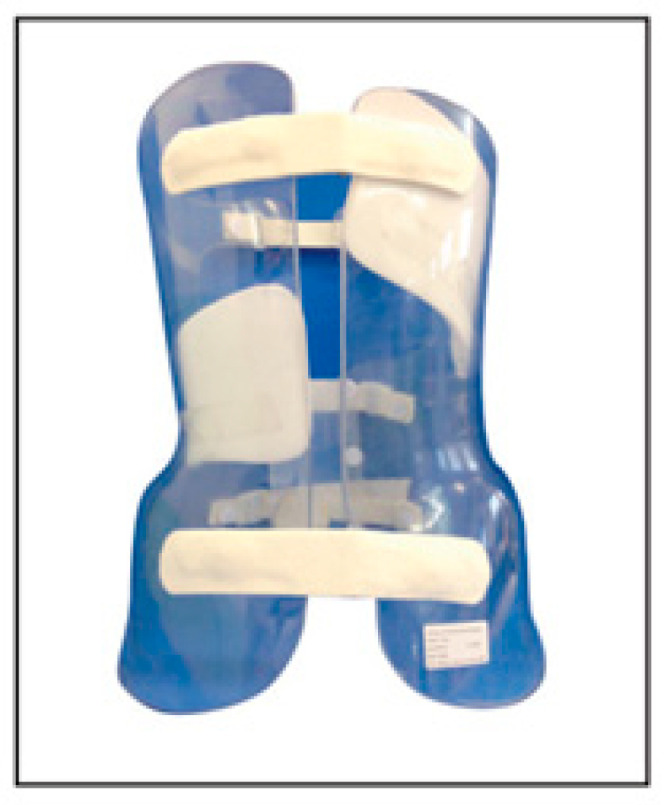
Sforzesco brace.

The SPoRT (symmetrical, patient-oriented, rigid, three-dimensional, active) is a new concept of bracing that follows specific principles of correction, and on this concept are based the Sibilla and Sforzesco braces [74,75]. These braces are custom-made according to the patient’s individual needs using technologies such as CAD-CAM that allow maximum customization of the orthosis [39]. The brace is then tested on the patient to modify and adapt it, depending on his or her real interaction between the body and the brace [75]. The Sforzesco brace (Figure 6), which is provided for free by the SSN, is formed by two lateral polycarbonate structures that envelop the trunk and connect along the posterior midline to a longitudinal metal rod. The correction takes place through three-dimensional elongation (a new concept different from the other corrective systems: 3-point, traction, postural, and movement-based) that pushes the spine longitudinally upwards, achieved through a system of thrusts on the major elevations and a creation of spaces on the pathological depressions [46,47]. CAD-CAM construction allows for a highly customizable prescription tailored to the patient’s curve, respecting the three planes, and allowing adjustments and customizations even in the control phase. Negrini et al., in 2011, identified a variation in time and duration of treatment depending on the severity from 18 to 23 h per day until Risser 3, then a gradual reduction. They also recommend the association with SEAS (Scientific Exercises Approach to Scoliosis) for the achievement of therapeutic success [74]. 

In a case/control and prospective cohort study conducted following both the SRS and SOSORT criteria by Negrini et al. [74], the Sforzesco brace was shown to be more effective than the Lyon brace and the Risser cast even when considered in association (Lyon brace plus Risser cast) in treating curves over 45 degrees Cobb. Furthermore, no patients showed a worsening beyond 45 Cobb degrees or underwent surgery, and an improvement in aesthetics was also observed [74].

A comprehensive summary of the key points of our overview is reported in Table 1.

## 4. Discussion and Conclusions

The aim of this review is to describe the braces provided by the Italian Health National Service for scoliosis treatment. The role of braces in scoliosis treatment is multifaceted and has been a subject of extensive research and clinical practice. Bracing has been found to be effective in preventing the progression of spinal curvature, particularly in adolescent idiopathic scoliosis. It aims to prevent spinal curve deterioration beyond a certain point, typically 45°, to avoid the need for surgery and preserve long-term quality of life. Bracing has also been shown to be effective in halting the progression of at-risk curves and, in some cases, even improving the Cobb angle. Furthermore, the use of braces can reduce thoracic hyperkyphosis in adolescents with scoliosis, demonstrating its impact on spinal alignment.

Despite a general consensus on the efficacy of rigid bracing treatment and its use in AIS, an important heterogeneity about the treatment is present in the scientific literature, demonstrating a high degree of variability [78,79].

The results of the studies included in this overview showed the superiority of the Boston and Cheneau braces over the Milwaukee braces, with Boston, Cheneau, and Lyon braces showing an overall high rate of success (range between 71% and >90%) in reducing curves less than 50 Cobb degrees. The efficacy of bracing increases with more time spent in the brace, so compliance is the key to treatment for both patients and parents [13,80,81]. It is also suggested to combine the brace with specific physiotherapy exercises for scoliosis (PSE) according to the SOSORT guidelines [24,82,83].The articles in the available scientific literature about the number of hours/day of bracing showed that the more hours per day the brace is worn, the better the result [30,83]. For this reason, braces are usually prescribed for at least 18 h/day, and they are usually removed for showers, swimming, physical education, and sports [84]. The patient should be stimulated to be active in sports and to continue to wear the brace if possible [30]. A combined treatment of bracing and physical activity was shown to be effective not only for correcting scoliotic curvature, but also to improve the antero-posterior and mediolateral body balance and to reduce the plantar load on the rearfoot region during gait, demonstrating effective mechanical action on the spine [85].

Unfortunately, there is a high rate of failure reported in the RCTs, which may be due to the difficulties found in performing RCTs in a field where parents reject randomization of their children, and this could impact the quality of the evidence. Usually, a curve evolution of six degrees or more during bracing treatment is considered a bad result, while progression to the need for surgical stabilization is considered a failure of brace treatment [30]. As suggested by Negrini et al., the predefined criteria identified by SRS and SOSORT could be used for the design of other studies, such as expertise-based trials or prospective controlled cohort studies, focusing on patient outcomes, adverse effects, the association between bracing treatment and PSSE, and patient compliance [7,86]. In the last few years, several studies have focused on how to increase patient compliance with bracing, and this step could be achieved using specific sensor monitoring [87]. 

In fact, while the effectiveness of bracing in the treatment of scoliosis has been well documented, challenges related to compliance and patient experience have also been noted. Rigid braces, although effective, have been described as uncomfortable, bulky, and aesthetically unappealing, leading to issues with compliance and psychological stress. Future directions in the employment of braces for scoliosis treatment are poised to be influenced by advancements in orthotic design, technology, and patient-centered approaches. The development of CAD/CAM-based brace models has shown promise in providing a classification-based approach to bracing scoliosis, offering superior in-brace correction and compliance compared to standard brace applications [88]. Additionally, the integration of 3D printing technology in the workflow of scoliosis brace adjustment presents significant potential for personalized and user-friendly brace design, enhancing the overall effectiveness of bracing treatment [89]. Furthermore, the use of very high-rigidity and high-precision CAD/CAM technologies has enabled the creation of corrective braces for adult scoliosis, indicating a shift towards more precise and tailored orthotic solutions [90].

The future of scoliosis bracing is also expected to be influenced by the digital world of artificial intelligence (AI), mathematical model calculations, and potentially 3D printing technology, reflecting a trend towards more technologically advanced and innovative approaches to brace therapy [91]. Moreover, the development of a novel robotic spinal brace for scoliosis treatment has been proposed as a potential solution to address the shortcomings of current braces, indicating a shift towards more advanced and sophisticated orthotic devices [37].

In addition to technological advancements, the future of bracing for scoliosis treatment is likely to be shaped by a focus on patient experience and quality of life. In fact, the success of any therapeutic intervention is profoundly influenced by the patient’s engagement and acceptance of the therapy. Active participation and a positive attitude towards the treatment significantly enhance its effectiveness, fostering better outcomes [92]. In this perspective, the experience of brace treatment in children/adolescents with scoliosis has been a subject of study, contributing to a better understanding of significant issues related to the experience of bracing therapy [93]. Particularly, stigmatization due to the orthopedic brace, restrictions on mobility, discomfort associated with its use, and the lack of acceptance among peers have been identified as significant problems affecting the effectiveness of treatment [94]. Furthermore, the psychological impact of brace use on children has become a major concern, highlighting the importance of considering the psychological and emotional aspects of brace treatment in scoliosis [95].

The strength of this overview is that, given the sound scientific evidence about their use, we can state that the spinal orthoses for AIS provided for free by the SSN (Milwaukee, Boston, Cheneau, Lyon, and rigid braces) arrest the progression of the scoliotic curve in the treatment of AIS. There is broad consensus on the approach to treatment and on the indications for the prescription of these spinal orthoses, including through tables and algorithms for assessing the risk of progression of the scoliotic curve. 

The main limitation of this overview is that only braces provided for free by the SSN were reported, so other kinds of braces (such as Providence or Charleston night-time braces) were not included. The braces reported in the present overview and provided for free by the SSN have solid scientific evidence to suggest their use. Further clinical trials are needed to provide unambiguous indications regarding the times of use, the clinical and radiological follow-up during treatment, the prevalent night or day use of the spinal orthosis, the timing, and methods of weaning from the brace.

## Figures and Tables

**Table 1:** A summary of the indication of bracing.

Which age group wears the braces more?	10–16 years [76]
Which brace is worn widely?	Boston brace [77]
What is the mean Cobb’s angle when a therapist suggests a particular brace for an individual?	40–45° Milwaukee, 20–50° Boston, 25–45° Cheneau, 30–50° Lyon, Sforzesco
How many hours the braces were worn per day?	23 h Milwaukee, 18–23 h Boston, 20–23 h Cheneau, 18–22 h Lyon, 18–23 h Sforzesco
Total treatment duration of wearing the brace.	2–4 years [30]
Success rates after wearing the braces?	48% (mean) Milwaukee, 72% Boston, 79% (mean) Cheneau, 88% (mean) Lyon, 78% (mean) Sforzesco

## Data Availability

Not applicable.
